# Re-analysis of metagenomic sequences from acute flaccid myelitis patients reveals alternatives to enterovirus D68 infection

**DOI:** 10.12688/f1000research.6743.2

**Published:** 2015-07-13

**Authors:** Florian P. Breitwieser, Carlos A. Pardo, Steven L. Salzberg

**Affiliations:** 1 Center for Computational Biology, McKusick-Nathans Institute of Genetic Medicine, Johns Hopkins School of Medicine, Baltimore, MD, 21205, USA; 2 Department of Neurology, Johns Hopkins Hospital, Baltimore, MD, 21205, USA; 3 Departments of Biomedical Engineering, Computer Science, and Biostatistics, Johns Hopkins University, Baltimore, MD, 21218, USA

**Keywords:** microbiome, metagenomics, neurological infections, computational biology, next-generation sequencing, sequence alignment

## Abstract

Metagenomic sequence data can be used to detect the presence of infectious viruses and bacteria, but normal microbial flora make this process challenging. We re-analyzed metagenomic RNA sequence data collected during a recent outbreak of acute flaccid myelitis (AFM), caused in some cases by infection with enterovirus D68. We found that among the patients whose symptoms were previously attributed to enterovirus D68, one patient had clear evidence of infection with *Haemophilus influenzae*, and a second patient had a severe *Staphylococcus aureus* infection caused by a methicillin-resistant strain. Neither of these bacteria were identified in the original study. These observations may have relevance in cases that present with flaccid paralysis because bacterial infections, co-infections or post-infection immune responses may trigger pathogenic processes that may present as poliomyelitis-like syndromes and may mimic AFM.  A separate finding was that large numbers of human sequences were present in each of the publicly released samples, although the original study reported that human sequences had been removed before deposition.

## Background

Metagenomic shotgun sequencing, in which DNA or RNA is extracted from a tissue sample and then sequenced, has the potential to detect a wide range of infections. Deep whole-genome shotgun (WGS) sequencing can detect bacteria, viruses, and eukaryotic pathogens with equal effectiveness, as long as the infectious agent is similar to a species that has been previously sequenced. Sequencing databases already contain thousands of known species, and as this number grows, the sensitivity of WGS will grow as well.

In 2014, a large outbreak of infection with enterovirus D68 was associated with both severe respiratory illness and acute paralysis, which the U.S. Centers for Disease Control and Prevention (CDC) named acute flaccid myelitis (AFM) ^1^. Samples collected from 48 patients were sequenced and shown to form a novel strain, Clade B1, based on phylogenetic analysis of 180 complete enterovirus D68 sequences ^2^. The same study conducted metagenomic sequencing of cerebrospinal fluid (CSF) and/or nasopharyngeal (NP) swabs from 22 of these patients and found enterovirus D68 in some NP samples that were positive based on PCR testing.

The identification of species from a WGS sample is a challenging problem that has spurred the development of multiple new computational methods ^3– 5^. Because of the large size of next-generation sequencing data sets, these methods need to be very fast, but in the context of clinical diagnosis, they also need to be accurate. We downloaded the 31 next-generation sequencing (NGS) samples from the Greninger *et al*. ^2^ study (NCBI accession SRP055445) and re-analyzed them using a computational pipeline based on the recently developed Kraken metagenomic analysis software ^4^, a very fast and sensitive system that can be customized to use a database containing any species whose sequences are available.

## Alternative infectious diagnoses in two subjects

Among the 22 subjects for which NGS data were available, we found at least two that had far greater numbers of sequences (reads) from a bacterial pathogen than from enterovirus D68. Neither subject had been reported in 2 as having a bacterial infection.

In one subject, US/CA/09-871, reported by Greninger *et al*. ^2^ as positive for enterovirus D68 through PCR and metagenomic NGS, we found in the NP swab sample an overwhelming presence of bacterial sequences from *Haemophilus influenzae*, a known cause of meningitis and neurological complications that was a common infection prior to the development of an effective vaccine.

Specifically, we identified 2,389,621 reads from *H. influenzae* in this subject, with the closest similarity to strain R2846. These reads comprise 93% of all microbial reads identified at the species level in the sample. Greninger *et al*. ^2^ reported 2,742 reads (in their Supplementary Table 4) matching enterovirus D68 ^2^ but did not report finding any *H. influenzae* reads from this sample. Our analysis found 1,330 reads matching enterovirus D68.

To confirm the identity of these reads, we aligned them separately to the complete genome of *H. influenzae* R2846, and we found that the reads completely covered the genome. Dividing the genome into 100 kilobase windows, depth of coverage varied from 266–828 reads/100Kbp, with far deeper coverage as expected at the 16S ribosomal RNA genes.

The enterovirus D68 isolated from patient US/CA/09-871 differed from the others in that it appeared in 2009, well before the 2014 outbreak, and that it grouped with Clade C, phylogenetically distinct from Clade B1 that was associated with AFM. This patient was reported ^2^ as having respiratory illness but not AFM. The sequence evidence here suggests that the patient might have had complications from *H. influenzae*-associated infection, although no clinical or CSF data was available for our re-analysis.

**Figure 1.**
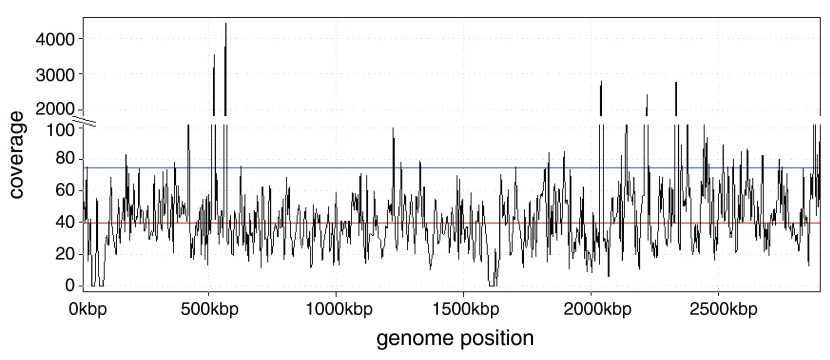
Depth of read coverage of the *S. aureus* MRSA252 genome using reads identified in the NGS sample from subject US/CA/12-5837. High peaks correspond to 16S rRNA genes. Red line: median coverage; blue line: mean coverage.

In a second subject, US/CA/12-5837, we found a strikingly large number of reads from *Staphylococcus aureus* in the NP swabs. The two separate NGS files associated with this subject contained 6,858,453 and 1,343,806 reads, comprising 70% and 84% (respectively) of all non-human reads identified at the species level in each sample. The closest match was *S. aureus* subsp. *aureus* MRSA252, a methicillin-resistant strain. The coverage was deep enough, approximately 40X, that it would be possible to assemble this genome separately from the reads here ( Figure 1). Greninger *et al*. ^2^ reported 2,790 reads from enterovirus D68 in this subject (our analysis found 1,641) but did not report any from *S. aureus*.

Patient US/CA/12-5837 was sampled in 2012, two years before the outbreak of AFM, although this patient was described in Greninger *et al*. ^2^ as positive for enterovirus D68 based on clinical PCR testing and metagenomic sequencing. This patient is reported to be one of the first patients with enterovirus-D68-positive AFM ^2^, but the sequence evidence indicates a severe *S. aureus* infection that might explain at least some of the patient’s symptoms. *S. aureus* has been implicated in neurological complications such as myelitis ^6^ and meningitis ^7^ by mechanisms that involve not only direct invasion into the central nervous system (CNS), but also immunopathogenic responses triggered by superantigens that can target the CNS ^8^. At a minimum, *S. aureus* infection was overlooked by the previous analysis. Although the potential role of bacterial infection in the neurological disease that affected these two subjects is difficult to assess because of the lack of clinical and CSF information, its involvement as a pathogenic co-factor should be evaluated.

## Human reads included in database submission

The metagenomics data (NCBI accession SRP055445) released by Greninger *et al*. ^2^ comprise 43 files which cover 22 of the 48 subjects from their study (in their Supplementary Table 1); the study did not conduct NGS for all subjects. Our metagenomics pipeline identifies human reads at the same time that it searches for pathogens; therefore we scanned the data for human as well as microbial content. Greninger *et al*. ^2^ reported that all human sequences had been removed from these files. We found, however, that all samples contained large numbers of human reads, ranging from a low of 18,215 to a high of 6,159,868. These comprised as few as 0.5% to as many as 95.6% of the reads in each sample, as shown in Table 1.

**Table 1.** Human reads found in metagenomic NGS samples from which human sequences were supposed to have been removed. Shown are the number of reads in each sample that clearly match the human genome and do not match any microbial species. AFM: acute flaccid myelits; NP: nasopharyngeal swap; CSF: cerebrospinal fluid.

Isolate	Run ID	Source	Number of human reads	%human
US/CA/12-5641	SRR1919640	NP	6,159,868	85.4
US/CA/12-5641	SRR1919641	NP	1,427,490	90.8
US/CA/12-5806	SRR1919642	NP	164,876	89.8
US/CA/12-5806	SRR1919643	CSF	202,677	95.5
US/CA/12-5807	SRR1919644	NP	160,719	94.1
US/CA/12-5807	SRR1919645	CSF	383,094	24.2
US/CA/12-5809	SRR1919646	NP	65,635	95.4
US/CA/12-5809	SRR1919647	NP	456,228	70.4
US/CA/12-5837	SRR1919648	NP	4,662,958	20.2
US/CA/12-5837	SRR1919649	NP	1,251,672	28.6
US/CA/14-5999	SRR1919650	CSF	3,046,664	89.9
US/CA/14-5999	SRR1919651	NP	1,407,842	71.0
US/CA/14-5999	SRR1919933	NP	174,140	68.5
US/CA/14-6000	SRR1919652	CSF	746,831	91.1
US/CA/14-6000	SRR1919653	NP	164,638	0.6
US/CA/14-6000	SRR1919934	NP	19,469	0.5
US/CA/14-6007	SRR1919654	CSF	352,391	85.4
US/CA/14-6010	SRR1919655	CSF	426,172	93.2
US/CA/14-6010	SRR1919656	NP	1,194,587	38.8
US/CA/14-6010	SRR1919935	NP	144,391	36.7
US/CA/14-6013	SRR1919657	NP	544,276	87.4
US/CA/14-6013	SRR1919658	NP	1,636,067	83.9
US/CA/14-6013	SRR1919936	NP	213,180	79.8
US/CA/14-6067	SRR1919659	CSF	567,263	3.9
US/CA/14-6067	SRR1919937	CSF	66,076	2.3
US/CA/14-6070	SRR1919660	CSF	578,579	4.3
US/CA/14-6070	SRR1919938	CSF	88,153	3.2
US/CA/14-6102	SRR1919661	CSF	791,143	82.4
US/CA/14-6102	SRR1919939	CSF	92,723	78.2
US/CO/13-60	SRR1919662	CSF	519,456	95.7
US/CO/13-60	SRR1919940	CSF	79,477	93.4
US/CO/14-86	SRR1919663	CSF	155,058	38.4
US/CO/14-86	SRR1919941	CSF	18,215	26.5
US/CO/14-88	SRR1919664	NP	453,411	3.8
US/CO/14-88	SRR1919942	CSF	39,899	2.7
US/CO/14-93	SRR1919665	CSF	758,650	96.6
US/CO/14-93	SRR1919943	CSF	123,250	95.3
US/CO/14-94	SRR1919666	NP	835,689	96.1
US/CO/14-94	SRR1919944	NP	131,998	95.2
US/CO/14-95	SRR1919667	CSF	352,679	2.8
US/CA/11-1767	SRR1919639	Culture	1,030,900	33.7
US/CA/10-786	SRR1919638	NP	130,044	0.5
US/CA/09-871	SRR1919637	CSF	384,285	11.0

The inclusion of human sequence data in the files deposited at NCBI was likely a result of a computational method (SURPI ^5^) that was insufficiently sensitive. Although the exact cause cannot be determined here, it is well known that sequence alignment algorithms often trade speed for sensitivity; e.g., by allowing fewer mismatches, an aligner can process reads at a much higher rate, at the cost of missing some alignments. It is less clear why the very large numbers of matches to two bacteria were missed; for both these bacteria, complete genomes from multiple strains are available in GenBank. We used both the Kraken system ^4^ and the Bowtie2 aligner ^9^ to ensure both sensitivity and speed in our analysis.

Release of sequence data is highly valuable, if not essential, for reproducibility and validation of sequencing-based studies. Failure to filter human reads from a sample is not uncommon; a recent study ^10^ found that Human Microbiome Project samples, from which human DNA was supposed to have been removed, contain up to 95% human sequence. This suggests that future efforts to deposit microbiome data need to employ more sensitive computational screens in order to avoid the unintentional release of human sequence data.

## Methods

Sequences were extracted from SRP055445 and each file was separately run through the Kraken program version 0.10.6-beta ( https://github.com/DerrickWood/kraken) ^4^, which identifies species by comparison with a database of all 31-bp sequences in all species. The database included the human genome (version GRCh38.p2), all complete bacterial and viral genomes, selected fungal pathogens, and known laboratory vector sequences from the NCBI UniVec database ( http://www.ncbi.nlm.nih.gov/tools/vecscreen/univec). Percentages of bacterial and viral reads in each sample were re-computed after excluding human and vector sequences. Reads matching more than one species were classified at the genus level or above. Reads from *H. influenzae* and *S. aureus* were re-aligned using Bowtie2 version 2.2.5 ^9^, a very fast and sensitive program for alignment of NGS reads to a reference genome, with the --local option. Bowtie2 was also used to re-align all reads from US/CA/12-5837 and US/CA/09-871 to the sequence of multiple enterovirus D68 strains (GenBank accessions JX101846.1, AY426531.1, KM851231.1, KM892500.1, KM892501.1, KM881710.2, KP745751.1, KP745755.1, KP745757.1, KP745760.1, KP745764.1, KP745766.1, and KP745767.1). We report the highest number of reads matching any one of these strains.
