# Supplementary material for: Re-analysis of metagenomic sequences from acute flaccid myelitis patients reveals alternatives to enterovirus D68 infection
Source: F1000Res. 2015 Jul 13;4:180. Originally published 2015 Jul 2. [Version 2] doi: 10.12688/f1000research.6743.2 (PMC4536609; doi:10.12688/f1000research.6743.2)
Supplement: Peer review history

# Peer review: Referee response for version 2

**DOI:** 10.5256/f1000research.7307.r9476

**Published:** 2015-08-12

**Authors:** Yoav Gilad

**Affiliations:** 1 Department of Human Genetics, University of Chicago, Chicago, IL, USA

The analysis is described in detail and the findings are clear. I found the comments of the authors of the original paper of interest, and the responses of Breitwieser *et al.* appropriate. I do think that it would be fair to mention in the abstract that this analysis does not challenge the main findings of the original paper. As currently written, the abstract alone does not make this point.

I have read this submission. I believe that I have an appropriate level of expertise to confirm that it is of an acceptable scientific standard.

# Peer review: Referee response for version 2

**DOI:** 10.5256/f1000research.7307.r9479

**Published:** 2015-07-15

**Authors:** David Lipman

**Affiliations:** 1 National Center for Biotechnology Information, National Library of Medicine, National Institutes of Health, Bethesda, MD, USA

This paper provides a straightforward reanalysis of the metagenomic data presented in Greninger *et. al*. and  does not contradict the basic interpretation of the results in that paper.  As noted in the exchange of comments on this paper by Greninger *et al.* and Breitwieser *et. al.,* while Greninger *et al*. noted the staph and h.flu sequences in the supplementary data, they did not note these in the paper itself or in the table in the supplementary data that provided additional details - despite the fact that these were present in very high proportions.  They also didn't report the presence of human sequence.  If all these data were presented explicitly and in the main body of Greninger *et al*., a reader would be more aware of the challenges of these metagenomic approaches in infectious disease.  I hope this paper will encourage this awareness and stimulate discussion so I note the comments on this paper from both sets of authors and I commend Breitwieser *et al.* for taking the time to respond as they have.

I have read this submission. I believe that I have an appropriate level of expertise to confirm that it is of an acceptable scientific standard.
